# New Skin-Relevant Cell Coculture Model With Stratum Corneum–Like Layer

**DOI:** 10.1155/2024/1041392

**Published:** 2024-06-19

**Authors:** Geovani Quijas, Agnieszka Lewińska, Marcin Łukaszewicz, Krzysztof Bojanowski

**Affiliations:** ^1^ Department of Dermatological Research Sunny BioDiscovery Inc., 972 E. Main Street, Santa Paula, California, USA; ^2^ Faculty of Chemistry University of Wroclaw, Joliot Curie 14 50-383, Wrocław, Poland; ^3^ Research Department OnlyBio Life S.A., 6 Jakóba Hechlińskiego 6 85-825, Bydgoszcz, Poland; ^4^ Faculty of Biotechnology University of Wrocław, Joliot Curie 14a 50-383, Wrocław, Poland; ^5^ Research and Development Department InventionBio S.A., Jakóba Hechlińskiego 4 85-825, Bydgoszcz, Poland

## Abstract

Two-dimensional (2D) cell culture is an important tool in the discovery of skin-active agents. Fibroblasts and keratinocytes, more rarely fibroblast–keratinocyte cocultures, are usually used for that purpose, where test compounds are added by mixing with the overlaying growth medium. However, such an approach is suboptimal because it lacks the *stratum corneum* component. The *stratum corneum* acts as a selective gatekeeper and opposes the intradermal permeation of many compounds that are bioactive when placed in direct contact with cells. One solution is to use reconstituted epidermis, but this approach is costly and time consuming. Here, a model is proposed, where the simplicity and convenience of the 2D cell culture is combined with the advantage of a hydrophobic barrier reminiscent of the skin horny layer. This model was tested with skin-relevant solvents, as well as with “naked” hydrophilic and encapsulated compounds. Cell viability and collagen stimulation were used as readouts. The results showed that the incorporation of a *stratum corneum*–substitute barrier on top of a 2D cell culture reduced the cytotoxicity of a common cosmetic solvent, dimethyl isosorbide (DMI), in cell culture and modified the bioactivity of the added actives (magnesium ascorbyl phosphate [MAP] and oligomeric proanthocyanidins [OPCs]/levan biopolymer), which became dependent on their ability to penetrate through a lipidic layer. Taken together, these results indicate a better physiological relevance of this cell culture model in workflows aimed at the discovery and analysis of skin-active compounds than conventional 2D systems.

## 1. Introduction

Since the pioneering experiments of Ross Harrison, Montrose Burrows, and Alexis Carrel in the first decade of the 20th century [1], cell culture has become a quasi-unavoidable technique in life sciences [2]. This is especially true in cosmetic science, where the use of animals for bioactivity testing is discouraged, and *in vitro* data often directly guide the design of human subject research and final product formulation. The most common cell culture systems utilized in dermatological and cosmetic research are two-dimensional (2D) monocultures of primary keratinocytes and fibroblasts. Their advantages include simplicity, broad availability, relatively low cost, and rapid result turnover. On the other hand, they do not represent an approximation of the skin, where fibroblasts and keratinocytes coexist in one organ, whose hydrophilic environment is protected by the hydrophobic cornified envelope.

To better approximate the physiology of the skin, three-dimensional (3D) laboratory–assembled models have been developed and commercialized [3–6]. They are composed of either an epidermis or an epidermis layered over a gelatinous matrix containing fibroblasts. These models are a big improvement over 2D monocultures, yet the latter is still the predominant standard in cell-based research focused on bioactivity. This is because 3D tissues are much more expensive and less amenable to high-throughput assay formats and take a few weeks to assemble. Furthermore, it is often challenging to perform direct or even indirect ELISAs (enzyme-linked immunosorbent assays) on them for structural proteins of particular interest in cosmetology and dermatology, such as collagens. Thus, there is a need for an intermediate design, which would retain most of the benefits but not the negatives of each model.

Here, we propose such a design, which combines 2D coculture of fibroblasts and keratinocytes in a 96-well plate format, with a *stratum corneum*–like hydrophobic barrier used in skin permeation assays called PAMPA (parallel artificial membrane permeability assay) [7] added to each well. Widely accepted as a screening tool for the approximation of the permeability of chemical entities through the cornified layer, PAMPA appears amenable to the combination with cell culture for the purpose of creating a more physiological model in bioactivity testing.

## 2. Materials and Methods

### 2.1. Cells

Normal human dermal fibroblasts (HDFs, ScienCell; Carlsbad, CA) and HaCaT keratinocytes (AddexBio; San Diego, CA) were cultured in DMEM supplemented with 5% FBS. For cocultures, both cell types were trypsinized, mixed 1:1, and plated at 10,000 cells per well in a 96-well cell culture plate.

### 2.2. Experimental Assay Setup

**Figure 1:**
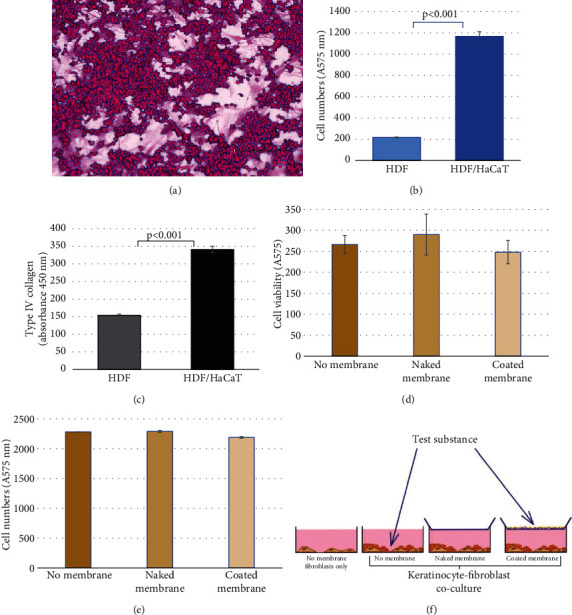
(a) Appearance of the 4-day-old HDF/HaCaT cell coculture after fixation and staining with sulforhodamine B (total magnification: 200x; bar: 150 *μ*m). The pink-stained larger fibroblasts are layered over by clusters of intensely colored smaller keratinocytes. (b) Comparison of cell numbers in HDF and HDF/HaCaT cells in 4-day-old cultures, showing that the dominant cellular components in cocultures are keratinocytes. (c) Comparison of type IV collagen level in HDF and HDF/HaCaT cell cocultures showing the statistically significant (*p* < 0.01) difference between both experimental conditions. Lack of statistically significant difference in cell viability (d) and cell numbers (e) between cocultures maintained without the superposed membrane, under an uncoated (naked) membrane or under phospholipid-coated membrane; (f) schematic illustration of the four culture types discussed here—HDF monoculture, HDF/HaCaT cell coculture, HDF/HaCaT cell coculture contacted with a hydrophobic membrane, and HDF/HaCaT cell coculture contacted with the phospholipid-coated hydrophobic membrane. A, absorbance. Error bars represent standard error of the mean. *p* values are calculated with unpaired *t*-test.

Cells were cultured in four experimental models: (A) HDF monoculture, (B) HDF/HaCaT cell coculture, (C) HDF/HaCaT cell coculture topped with a hydrophobic PVDF membrane (Millipore, Burlington, MA, cat.# MAIPNTR10, pore size 0.45 *μ*m) contacting the cell culture medium, and (D) HDF/HaCaT cell coculture superposed with the same membrane coated with 1% phospholipid (L-*α*-phosphatidylcholine, a natural phospholipid, Sigma, St. Louis, MO, cat.# 3644) in dodecane (Figure 1(f)).

Two cosmetic substances were tested: the known hydrophilic collagen stimulator magnesium ascorbyl phosphate (MAP, Sigma, St Louis, MO, cat.# A8960) and SBD.5HCL, which was prepared by the encapsulation of oligomeric proanthocyanidins (OPCs; 95% *w*/*w*) in a 3% solution of nanocapsules made of a microbial levan biopolymer and acting as transdermal carrier +1.5% Euxyl K903 (a preservative), as described before [8, 9]. The use of SBD.5HCL has been guided by the long-term interest of our lab in the intrategumental delivery of OPC [10, 11]. Both substances were tested either by mixing directly with the cell culture medium or placing them on top of a phospholipid-coated PVDF membrane in the 96-well plate format, as illustrated in Figure 1(f).

Furthermore, dimethyl isosorbide (DMI), a common cosmetic solvent, was assayed to compare its cytotoxicity, when mixed directly with the cell culture medium to the application on top of a phospholipid-coated membrane barrier.

### 2.3. Experimental Endpoints

The experimental endpoints assessed after 4 days of incubation were (1) cell numbers quantified with the standard sulforhodamine B method [12], (2) cellular metabolism using the standard resazurin (Alamar Blue) reduction assay [13], and (3) type IV collagen in formalin-fixed cell cultures assayed by direct ELISA using the anticollagen IV biotinylated antibody cat. #1340-08 from SouthernBiotech (Birmingham, AL) streptavidin-HRP and TMB reagents (BioLegend, San Diego, CA). Cells were observed, and microphotographs were acquired with an Evos 5000 imaging system (Thermo Fisher Scientific, Waltham, MA). Colorimetric signals were quantified on the multimode SpectraMax i3x platform (Molecular Devices, San Jose, CA) with SoftMax Pro 7.0.3 software and reported in arbitrary colorimetric absorbance units.

### 2.4. Statistical Analysis

*p* values representing statistical significance were calculated using an unpaired two-tailed *t*-test (Student's method), and the threshold of statistical significance was fixed at *p* = 0.05 and 15% difference compared to the water control group.

## 3. Results

### 3.1. Validation of the Coculture Model

The cocultures of normal human fibroblasts and HaCaT keratinocytes remained viable for at least 4 days (Figure 1(a)). These cocultures recorded a much higher cell numbers than fibroblast monocultures at Day 4, showing that, like in the skin, keratinocytes were the main proliferating component (Figure 1(b)). The type IV collagen level was significantly higher in cocultures than in fibroblast monocultures (Figure 1(c)), which is consistent with the increased metabolism in cocultures and points to a possible cross-talk between these two skin cell types, not unlike in organotypic 3D systems [14]. The metabolic activity (Figure 1(d)) and cell numbers (Figure 1(e)) in the cocultures were not affected by the contact with the hydrophobic PDVF membrane regardless of the inclusion of the *stratum corneum*–like phospholipid layer. Figure 1(f) illustrates various experimental setups employed herein.

### 3.2. Testing Two Types of Compounds for Bioactivity in the Coculture Model With or Without the Barrier

**Figure 2:**
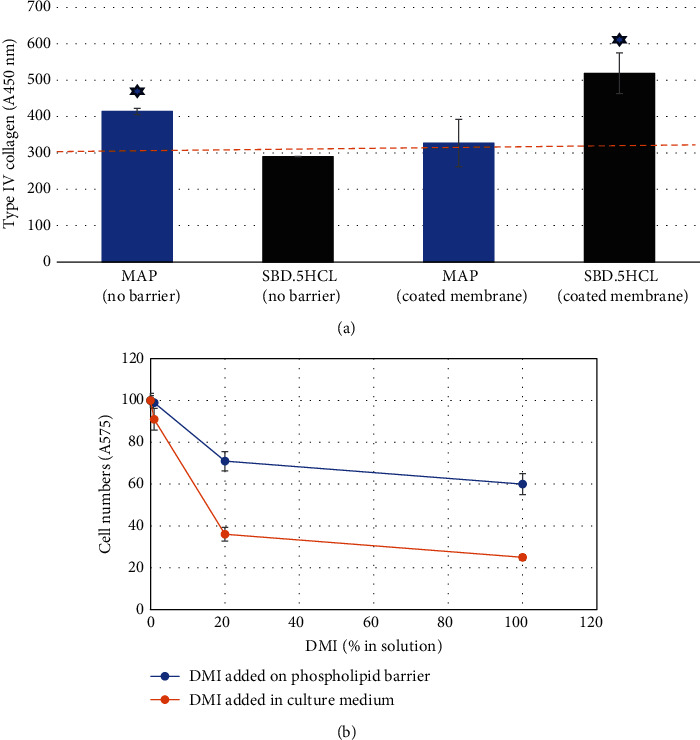
Proof of concept of the phospholipid-lined membrane as a *stratum corneum*–like barrier for dermatological and cosmetic active testing. (a) MAP stimulates type IV collagen output in the HDF/HaCaT coculture by over 30% when added directly (no barrier) to the cell culture medium, but not when placed on top of the phospholipid-coated membrane. In contrast, the addition to same membrane of the compound designed to penetrate through the outer layer of the skin (SBD.5HCL) results in over 60% collagen upregulation, although it contains a preservative. Same preserved test material is inactive when added directly (no barrier) to the cell culture conditioned medium. The horizontal dotted line represents the level of type IV collagen in water-treated negative controls. A450nm, absorbance at 450 nm expressed in arbitrary colorimetric units. Asterix, collagen stimulation by MAP in (no barrier) and by SBD.5HCL in (coated membrane) experimental conditions is statistically significant versus water-treated control (*p* < 0.01). (b) A common cosmetic solvent DMI is less toxic when added on a phospholipid-coated membrane that when mixed directly with the cell culture medium, showing more physiologically relevant response to the former approach. A575nm, absorbance at 575 nm expressed in arbitrary colorimetric units. The difference between cytotoxic effect in barrier versus no-barrier experimental conditions is statistically significant for 100% DMI and 20% DMI. Error bars represent standard error of the mean. *p* values are calculated with unpaired *t*-test.

The hydrophilic collagen stimulator MAP expectantly [15] upregulated type IV collagen in HDF-HaCaT cell cocultures when added directly to the medium; however, such stimulation was not detected when MAP was added to phospholipid-coated membranes over HDF/HaCaT cell cultures. In contrast, the encapsulated compound SBD.5HCL formulated to penetrate the horny layer had no effect when added directly to the HDF/HaCaT cell cocultures but triggered a statistically significant upregulation of type IV collagen when placed on top of the phospholipid-lined PVDF membrane contacting the cocultures (Figure 2(a)).

### 3.3. Testing the Cytotoxicity of DMI in the Coculture Model With or Without the Barrier

DMI is a common solvent used in cosmetic formulations approved for skin application. However, it is highly cytotoxic when introduced directly to the cell culture medium. Applying it, instead, on the barrier of a phospholipid-coated membrane allows to substantially lower its cytotoxic effect, thus bringing testing conditions closer to the physiological state (Figure 2(b)).

## 4. Discussion

Cell culture is one of the key tools for assessing the bioactivity and safety of dermatological and cosmetic products. Mainly 2D monocultures of fibroblasts and keratinocytes are employed; more rarely, fibroblast/keratinocyte cocultures are used [16]. Here, we utilized such a coculture system to which we added a hydrophobic PVDF membrane coated with a phospholipid. Such membranes have been extensively used to investigate the passive diffusion of skin actives, including the cosmetic ones, in PAMPAs [7, 17, 18]. However, to the best of our knowledge, this is the first time that the PAMPA was combined with life cell culture and used as an artificial *stratum corneum*–like barrier for bioactivity/cytotoxicity cell-based assays. This model has obvious limitations, such as lack of an ordered 3D epidermal tissue structure, use of an immortalized HaCaT line carrying gain-of-function mutations in p53, an artificial membrane coated with a simple phospholipid instead of the complete *stratum corneum* make-up, and even pH limitations [19]. Another limitation of this approach is the inability to readily distinguishing between the effects on fibroblasts and keratinocytes in coculture, although components specific to only one of these cell types, such as collagens or keratins, can be quantified by ELISA or qPCR with specific probes. These limitations were dictated by the quest for simplicity: HaCaT cells can be easily cultured in the same medium as fibroblasts, which is more complicated in the case of normal human keratinocytes, although certainly feasible in the proposed model, as well.

Despite the abovementioned limitations, the model presented here demonstrated its usefulness in several areas. One of them is the more physiologically relevant placement of the tested sample—on top of a phospholipid barrier, instead of in direct contact with skin cells. As illustrated in Figure 2(a), such placement voids the bioactivity of an ascorbic acid derivative, which is a strong collagen inducer when applied directly to cells. Another improvement is the ability to test samples which contain preservatives. Testing preserved materials in 2D cultures is challenging because the preservatives inhibit cellular metabolism when placed in direct contact with cells. Here, this is illustrated by the fact that the Euxyl-preserved formulation of SBD.5HCL is effective in stimulating collagen when placed atop the phospholipid barrier but not when it is added to the cell growth medium (Figure 2(a)). Yet another advantage of this model is the ability to test substances in solvents, which would have been cytotoxic when added directly to the cell culture medium (Figure 2(b)).

The phospholipid-coated PVDF membrane is an inexpensive, uncomplicated, and well-published substitute for the horny layer and is accessible to any life sciences laboratory. The model can be adapted to accommodate specific needs—melanocytes can be added to the mix, the ratio of keratinocytes to fibroblasts can be modulated, or a cellular component of the coculture may be omitted (HaCaT cells seem to diminish the metabolic activity of HDFs). Likewise, the simple phospholipid coating of the membrane may be replaced by a more sophisticated assembly of *stratum corneum* ingredients [20]. Either way, the shielding of the top of the well should result in decreased evaporation, especially in the peripheral wells, making the bioactivity measurements more uniform. Finally, this system can be adapted to study the effect of test substances on enzymatic systems in vitro by just replacing cell culture with a specific reaction mixture. In summary, the proposed barrier-inclusive model is a simple, adaptable yet robust upgrade to the traditional approach for testing skin-active agents and can be incorporated into every cell-based R&D workflow. It can be used to test either isolated actives or finished formulas that are otherwise incompatible with the cell culture medium and can be expanded to investigate the effects of topically applied substances on enzymatic systems.

## Data Availability

Data is available on request. The request should be addressed to the corresponding author (Dr. Krzysztof Bojanowski) by email (kbojanowski@sunnybiodiscovery.com) or telephone (+1 805 229 7580).
